# Association of Serum Secreted Frizzled-Related Protein 5 Levels With Coronary Artery Disease

**DOI:** 10.7759/cureus.80408

**Published:** 2025-03-11

**Authors:** Sophia Selvakumari J, M Saradha, K Kayalvizhi, Praveena Daya A, Christal Viji Shalini

**Affiliations:** 1 Biochemistry, All India Institute of Medical Sciences, Madurai, Madurai, IND; 2 Biochemistry, Tirunelveli Medical College, Tirunelveli, IND; 3 Biochemistry, Government Medical College, Virudhunagar, Virudhunagar, IND; 4 Community and Family Medicine, All India Institute of Medical Sciences, Madurai, Madurai, IND

**Keywords:** adipokine, anti-inflammatory, coronary artery disease (cad), secreted frizzled-related protein 5 (sfrp5), wnt 5a signaling

## Abstract

Background

Coronary artery disease (CAD) is the leading cause of death and premature disability worldwide. Secreted frizzled-related protein 5 (SFRP5), a recently identified adipokine, acts as an antagonist of Wingless-type family member 5A (WNT5A) signaling. Since WNT5A triggers inflammation in endothelial cells, the anti-inflammatory properties of SFRP5 play a crucial role in counteracting this effect. Lower SFRP5 levels contribute to the chronic inflammatory conditions associated with CAD by promoting WNT5A signaling.

Aim

This study aimed to assess serum SFRP5 levels in CAD patients and compare them with those in non-CAD patients at a tertiary care hospital.

Materials and methods

The study sample included 40 angiographically confirmed CAD patients as cases and 40 non-CAD patients as controls. Serum SFRP5 levels and lipid profiles were measured and compared between the two groups. Data analysis was performed using IBM SPSS Statistics for Windows, Version 26.0 (Released 2019; IBM Corp., Armonk, NY, USA), employing descriptive statistics, normality tests, t-tests, chi-square tests, and correlation analyses. A p-value of <0.05 was considered statistically significant.

Results

Serum SFRP5 levels were significantly lower in CAD patients (1.25 ± 0.35) compared to controls (2.46 ± 0.96) (p < 0.01). Additionally, SFRP5 showed a significant negative correlation with triglycerides and low-density lipoprotein cholesterol (p < 0.05).

Conclusions

This study demonstrated that serum SFRP5 levels were significantly lower in CAD patients compared to controls. Therefore, serum SFRP5 may serve as a novel biomarker for the early prediction of CAD.

## Introduction

In the 21st century, as the world has triumphed over infectious diseases, a rapid epidemiological shift toward noncommunicable diseases (NCDs) has emerged, posing a significant challenge to healthcare systems. NCDs account for 71% (41 million) of the 57 million global deaths [1], with cardiovascular diseases (CVDs) responsible for 19.1 million deaths. Coronary artery disease (CAD) is the most prevalent CVD, affecting a staggering 244.1 million people worldwide [2].

In India, the proportion of total deaths attributed to NCDs rose from 37.9% in 1990 to 61.8% in 2016 [3]. Among these, CAD was the leading cause of mortality, with a death rate twice as high as the next leading cause. The increasing prevalence of CAD in India is largely driven by lifestyle changes, urbanization, and industrialization. Early detection of CAD is therefore crucial in mitigating its severe impact. In this pursuit, the relentless search for novel biomarkers offers a promising avenue for earlier diagnosis and prevention, ultimately improving cardiovascular health. Among the emerging biomarkers, secreted frizzled-related protein 5 (SFRP5) has recently garnered significant attention.

Studies suggest that WNT signaling plays a crucial role in the development of atherosclerosis. The term “WNT,” coined by Nusse and Varmus in 1982 [4], originates from a combination of the *Drosophila *gene *WINGLESS *and the homologous mouse proto-oncogene *INT-1*. Activation of WNT5A has been linked to endothelial dysfunction, inflammation, and vascular smooth muscle cell proliferation [5]. SFRP5, a novel anti-inflammatory adipokine, has been proposed to bind to WNT5A, thereby blocking WNT signaling pathways [6]. Given its antagonistic role, SFRP5 is believed to have cardioprotective properties.

Based on these findings, we hypothesized that CAD patients would exhibit lower serum SFRP5 levels. While existing evidence highlights the significance of SFRP5 in CAD, studies investigating its association with CAD in the Indian population remain limited. This study aims to assess serum SFRP5 levels in CAD patients, compare them with controls, and determine whether SFRP5 could serve as a potential biomarker for CAD diagnosis.

Objective

The primary objective was to estimate and compare serum SFRP5 levels in patients with and without CAD at a tertiary care hospital. The secondary objective was to examine the correlation between serum SFRP5 levels and cardiometabolic risk factors.

## Materials and methods

This case-control study was conducted at Tirunelveli Medical College in Tirunelveli, India, from December 2019 to December 2020, following approval from the Institutional Research Ethics Committee (ref. no. 1421/Bio/2018, dated November 30, 2018).

Sample size and sampling technique

Based on SFRP5 levels reported in a study by Gharibi et al. [7] - mean ± SD: 28.60 ± 8.91 in cases and 39.92 ± 17.09 in controls - the required sample size was estimated using OpenEpi software version 3. With 90% power and a 95% confidence level, the calculated sample size was 31 cases and 31 controls. To account for a 20% nonresponse rate, the final sample size was rounded to 40 cases and 40 controls.

Study population

Cases included 40 angiographically confirmed CAD patients with more than 50% stenosis in at least one major coronary artery, recruited from the Department of Cardiology. Patients with acute or chronic inflammatory diseases, stroke, heart failure, recent myocardial infarction (within the past three months), or recent surgeries (within the past three months) were excluded. The control group consisted of 40 patients who underwent coronary angiography and were found to be free of CAD. Age, sex, and confounding factors such as diabetes, hypertension, smoking, and alcohol consumption were matched between cases and controls. Written informed consent was obtained from all participants before enrollment.

Study tool

A semi-structured questionnaire was used to collect sociodemographic data, including age, gender, height, and weight, along with clinical and biochemical parameters. Recumbent blood pressure and a 12-lead ECG were recorded for each participant. After an eight- to 12-hour fasting period, 5 mL of venous blood was collected from each participant. The lipid profile was measured immediately after serum separation using a fully automated analyzer. Serum SFRP5 levels were estimated using the ELK3135 Human SFRP5 Enzyme Immunoassay Kit (Wuhan, China). Low-density lipoprotein (LDL) cholesterol (LDL-c) was calculated using the Friedewald formula:

\[ \text{LDL-c} = \text{TC} - (\text{HDL} + \text{VLDL}) \]

where

\[ \text{VLDL-c} = \frac{\text{TGL}}{5} \]

Statistical analysis

Data were entered into Microsoft Excel 2016 (Microsoft Corporation, Redmond, WA, USA) and analyzed using IBM SPSS Statistics for Windows, Version 26.0 (Released 2019; IBM Corp., Armonk, NY, USA). Categorical variables were expressed as frequencies and percentages. The normality of continuous variables was assessed using the Shapiro-Wilk test. Normally distributed continuous variables were presented as mean ± SD, and comparisons between groups were conducted using an independent sample t-test. Associations between categorical variables were analyzed using the chi-square test. The correlation between serum SFRP5 levels and other relevant parameters was assessed using Pearson’s correlation test. A p-value of <0.05 was considered statistically significant for all analyses.

## Results

This study included 80 participants, comprising 40 CAD cases and 40 matched controls without CAD. The demographic and clinical profiles of the study participants are summarized in Table 1. The mean age of cases was 56.42 ± 10.29 years, while that of controls was 57.07 ± 10.35 years, with no statistically significant difference between the groups. Additionally, potential confounding factors such as diabetes, hypertension, smoking, and alcohol use were matched, and no significant differences were observed.

**Table 1:** Demographic and clinical profile of study participants (n = 80) ^*^ p-value < 0.05 was considered statistically significant.

Variables	Cases (n = 40)		Controls (n = 40)		X² value	p-Value^*^
	N	%	N	%		
Gender						
Male	34	85	30	75	1.25	0.264
Female	6	15	10	25		
Presence of diabetes	7	17.5	9	22.5	0.31	0.576
Presence of hypertension	13	32.5	14	35	0.56	0.813
Smoking	13	32.5	15	37.5	0.22	0.639
Alcohol intake	10	25	13	32.5	0.54	0.459

The mean SFRP5 levels in cases and controls are presented in Table 2. Cases had a lower mean SFRP5 level (1.25 ± 0.35) compared to controls (2.46 ± 0.96), and this difference was statistically significant.

**Table 2:** Distribution of mean SFRP5 among cases and controls SFRP5, secreted frizzled-related protein 5

SFRP5 (ng/mL)	Mean ± SD	95% CI (lower to upper)	t-Value	p-Value
Cases (n = 40)	1.25 ± 0.35	-1.508 to -0.792	6.44	<0.001
Controls (n = 40)	2.46 ± 0.96			

The mean values of various biochemical parameters were compared between cases and controls, as presented in Table 3. Statistically significant differences were observed between the groups for total cholesterol (TC), triglycerides (TGL), very low-density lipoprotein, and LDL levels.

**Table 3:** Comparison of mean cardiometabolic risk factors between cases (n = 40) and controls (n = 40) ^*^ An independent sample t-test was used to compare the means, and a p-value < 0.05 was considered statistically significant. HDL, high-density lipoprotein; LDL, low-density lipoprotein; TC, total cholesterol; TGL, triglycerides; VLDL, very low-density lipoprotein

Biochemical parameters	Cases (mean ± SD)	Controls (mean ± SD)	Mean difference	95% CI (lower to upper)	t-Value	p-Value^*^
TC	188.33 ± 43.18	154.18 ± 29.8	34.15	17.594 to 50.706	4.114	<0.001
TGL	178.78 ± 48.9	136.5 ± 30.54	42.275	24.071 to 60.479	4.637	<0.001
HDL	39.15 ± 5.54	40.25 ± 6.29	-1.1	-3.741 to 1.541	-0.829	0.409
VLDL	35.80 ± 9.77	27.28 ± 6.06	8.525	4.892 to 12.158	4.686	<0.001
LDL	113.38 ± 37.31	86.65 ± 26.75	26.725	12.272 to 41.178	3.681	<0.001

Table 4 presents the comparison of mean SFRP5 levels between the two groups based on gender and BMI. No significant differences were observed between the groups for either factor.

**Table 4:** Comparison of SFRP5 levels between cases and controls based on gender and BMI SFRP5, secreted frizzled-related protein 5

Variable	N	Mean± SD	p-Value
Gender			
Male	64	1.93 ±0.83	0.64
Female	16	2.07 ± 1.15	
BMI			
BMI <25	43	1.93 ± 0.97	0.45
BMI >25	37	1.77 ± 0.90	

Table 5 presents the Pearson correlation between SFRP5 and cardiometabolic risk factors. A statistically significant negative correlation was observed between SFRP5 and TC, TGL, and LDL.

**Table 5:** Pearson correlation of SFRP5 with cardiometabolic risk factors ^*^ Correlation is significant at the 0.01 level (two-tailed). HDL, high-density lipoprotein; LDL, low-density lipoprotein; SFRP5, secreted frizzled-related protein 5; TC, total cholesterol; TGL, triglycerides

Biochemical parameters	Pearson correlation	p-Value	Interpretation
TC	-0.512^*^	<0.001	Moderate negative correlation, significant
TGL	-0.518^*^	<0.001	Moderate negative correlation, significant
HDL	0.109	0.334	Not significant
LDL	-0.446^*^	<0.001	Moderate negative correlation, significant

## Discussion

In this study, serum SFRP5 levels were measured and compared between patients with CAD and those without. The findings provide additional evidence supporting an association between CAD and the anti-inflammatory adipokine SFRP5. Compared to healthy controls, CAD patients exhibited significantly lower serum SFRP5 levels, aligning with studies by Gharibi et al. [7] and Miyoshi et al. [8].

SFRP5 exerts its anti-inflammatory effects by suppressing Wingless-type family member 5A/c-Jun N-terminal kinase (WNT5A/JNK) signaling in adipose tissue and macrophages [9]. Over the past decade, growing evidence has highlighted the role of WNT signaling in the pathophysiology of atherosclerosis. Studies have demonstrated elevated WNT5A expression in both murine and human atherosclerotic lesions [10]. Further evidence from Tsaousi et al. suggests a direct link between WNT signaling and atherogenesis, emphasizing its involvement in various stages of atherosclerosis, from endothelial dysfunction to vascular remodeling post-myocardial infarction [11]. Similarly, research by Gay and Towler has illustrated how WNT signaling contributes to metabolic dysfunction and structural cardiac changes, including plaque formation [12]. Another study found that elevated WNT5A levels in endothelial cells of diabetic patients led to increased JNK activation, resulting in endothelial dysfunction [13].

This crucial pathway is tightly regulated by extracellular antagonists such as WNT inhibitory factor 1, Dickkopfs, and SFRPs, highlighting the potential for therapeutic intervention [6]. SFRPs, a family of five glycoproteins in humans (SFRP1-5), serve as negative regulators of WNT signaling [14]. Notably, SFRP5 functions as an endogenous extracellular inhibitor of WNT5A signaling, underscoring its critical role in CAD. It is expressed in the endocardium, epicardium, pericardium, and all myocardial chambers except the right ventricle [15]. Additionally, SFRP5 has been shown to suppress inflammatory responses following ischemic injury in the heart by antagonizing WNT5A signaling. Nakamura et al. demonstrated that genetic deficiency of SFRP5 in mice resulted in larger myocardial infarct sizes after ischemia/reperfusion injury, increased apoptotic cardiac myocyte death, and heightened inflammation in the infarct zone [9].

The protective role of SFRP5 extends to arterial aging as well. Teliewubai et al.’s study showed that SFRP5 inhibits vascular smooth muscle cell proliferation, migration, and inflammation, which was demonstrated in vitro by its suppression of platelet-derived growth factor-induced rat aortic smooth muscle cell proliferation and migration [16]. Furthermore, Cho et al. demonstrated that SFRP5 exerts vasorelaxant effects by enhancing endothelial nitric oxide function through inhibition of the WNT5A/JNK signaling pathway [17].

Given that adipokine levels can vary between genders, the relationship between SFRP5 and sex was examined. No significant difference in SFRP5 levels was observed between male and female participants (p = 0.64), as shown in Table 4. This finding is consistent with Hu et al.’s study [18]. Although this study did not find a statistically significant gender difference in SFRP5 levels, potential differences could still exist, potentially influenced by genetic, environmental, and lifestyle factors.

The relationship between SFRP5 and obesity remains complex, with studies yielding inconsistent results. Some research has found no significant difference in SFRP5 levels between normal weight and obese individuals, while others suggest lower SFRP5 levels in obesity. In this study, no significant difference in mean SFRP5 levels was observed between normal weight and obese individuals, consistent with Schulte et al.’s findings, which suggested that SFRP5 levels do not directly correlate with obesity but increase with calorie restriction [19]. Xu et al.’s study yielded similar results [20]. However, Hu et al. and Rydzewska et al. reported decreased SFRP5 levels in obese individuals with type 2 diabetes [18,21].

Dyslipidemia is a key driver of atherosclerosis, and as an anti-inflammatory adipokine, SFRP5 is hypothesized to regulate lipid metabolism and mitigate the risk of CAD. In this study, serum SFRP5 levels were negatively correlated with TC, TGL, and LDL, with a weak positive correlation observed with HDL. These findings align with previous studies exploring the link between SFRP5 and lipid metabolism [22]. The observed reduction in SFRP5 levels in individuals with dyslipidemia may be a consequence of chronic inflammation. Given this association, SFRP5 could serve as a biomarker for cardiovascular risk stratification in patients with dyslipidemia.

Recent studies suggest that recombinant SFRP5 can reduce ischemic myocardial injury, highlighting its potential as a novel therapeutic target for CAD treatment [23].

In light of these findings, larger studies are needed to validate these results in broader populations. Additionally, interventional studies are required to evaluate the therapeutic potential of SFRP5 in CAD.

Limitations

The design of this study limits the ability to establish a causal relationship between serum SFRP5 levels and the development of CAD. Additionally, serum WNT5A levels were not measured, which could have provided further insights and strengthened the study’s validity.

## Conclusions

This study highlights the indispensable role of SFRP5 in the pathogenesis of CAD. Over the past two decades, extensive research has firmly established the activation of WNT signaling in atherosclerosis, contributing to endothelial dysfunction, inflammatory responses, and vascular smooth muscle cell proliferation. As a novel anti-inflammatory adipokine, SFRP5 has been proposed to bind to WNT5A, thereby blocking WNT signaling pathways. This inhibition reduces chronic inflammation and exerts a protective effect on the vascular endothelium, a finding supported by the current study.

Based on these findings, SFRP5 levels could serve as a surrogate marker for CAD, enabling early intervention and potentially improving patient outcomes.
